# Adenine-induced chronic kidney disease induces a similar skeletal phenotype in male and female C57BL/6 mice with more severe deficits in cortical bone properties of male mice

**DOI:** 10.1371/journal.pone.0250438

**Published:** 2021-04-23

**Authors:** Corinne E. Metzger, Elizabeth A. Swallow, Alexander J. Stacy, Matthew R. Allen

**Affiliations:** 1 Department of Anatomy, Cell Biology & Physiology, Indiana University School of Medicine, Indianapolis, IN, United States of America; 2 Department of Medicine, Division of Nephrology, Indiana University School of Medicine, Indianapolis, IN, United States of America; 3 Department of Biomedical Engineering, Indiana University Purdue University of Indianapolis, Indianapolis, IN, United States of America; 4 Roudebush Veterans Administration Medical Center, Indianapolis, IN, United States of America; University of Life Sciences in Lublin, POLAND

## Abstract

Chronic kidney disease (CKD) causes bone loss, particularly in cortical bone, through formation of cortical pores which lead to skeletal fragility. Animal models of CKD have shown variability in the skeletal response to CKD between males and females suggesting sex may play a role in this variation. Our aim was to compare the impact of adenine-induced CKD on cortical parameters in skeletally mature male and female C57Bl/6 mice. After 10-weeks of adenine-induced CKD, both male and female adenine mice had high serum parathyroid hormone (PTH), high bone turnover, and cortical porosity compared to non-CKD controls. Both sexes had lower cortical thickness, but only male mice had lower cortical bone area. CKD imparted greater deficits in mechanical properties of male mice compared to female mice. These data demonstrate that both male and female mice develop high PTH/high bone turnover in response to adenine-induced CKD and that cortical bone phenotypes are slightly more severe in males, particularly in mechanical properties deficits.

## Introduction

Bone loss is largely due to cortical bone deterioration in the form of cortical porosity and is common in chronic kidney disease (CKD) patients. Average annual changes in cortical porosity in CKD patients at the distal tibia and distal radius changed more drastically (~4% increase) than other cortical or trabecular parameters [1]. Not surprisingly, skeletal fragility is a common comorbidity of CKD with hip fracture incidence higher in CKD patients compared to those with normal kidney function [2] and even higher in patients requiring hemodialysis [3, 4]. Irrespective of disease severity, CKD patients experience higher rates of post-fracture complications, hospitalization, and mortality than the non-CKD population [2, 5, 6]. There is an imperative need to better understand aspects of CKD-induced bone porosity and fragility.

CKD impacts both males and females with a slightly greater prevalence in women than in men in the United States [7, 8] and worldwide [9]; however, not all animal models of CKD allow for direct sex comparisons. For example, male Cy/+ rats with a genetic progressive kidney disease, develop high parathyroid hormone (PTH), vascular calcifications, and cortical porosity [10, 11], but females do not develop disease phenotypes even with ovariectomy [12]. Also in rats, both adenine-induced CKD and CKD induced by chronic nitric oxide inhibition result in more severe disease phenotype in male vs. female rats [13, 14]. The impact of sex on CKD-induced cortical porosity and skeletal phenotypes remains largely unknown. Since CKD impacts both sexes, animal models that allow for direct sex comparisons are important to help understand the pathophysiology and assess potential therapeutics.

In this current study we aimed to compare the bone phenotype produced from adenine-induced CKD in male and female C57Bl/6J mice. We hypothesized that male mice with adenine-induced CKD would have a significantly worse skeletal phenotype, including higher cortical porosity and lower mechanical properties than female adenine mice.

## Materials and methods

### Animals

Male and female C57Bl/6J mice (B6; JAX #000664; n = 16 per sex) were ordered from Jackson Laboratories (Bar Harbor, ME, USA) at 15 weeks of age and group housed four per cage at an institutionally approved animal facility with 12-hour light/dark cycles with an average temperature of 70°F. All cages of mice were provided with nest building material (crinkle paper) for enrichment. At 16 weeks of age, all mice were switched to a purified casein-based diet with adjusted calcium and phosphorous (0.9% phosphorous, 0.6% calcium) and half of the mice in each group (n = 8) were given an addition of 0.2% adenine in the diet (Envigo Teklad Diets, Madison, WI, USA). After a 6-week induction on the adenine diet, all adenine mice were switched back to the control casein-based diet for four weeks as previously described [15]. Mice were monitored daily, and body weights and food intake were measured weekly throughout the protocol. All mice were injected with fluorochrome calcein labels 7 and 2 days prior to euthanasia. All mice made it to study end point. After 10 weeks on diets, animals were anesthetized via vaporized inhaled isoflurane and euthanized via exsanguination and thoracotomy. Kidney, right femurs, and right humeri were fixed in 10% neutral buffered formalin for 48 hours and then stored in 70% ethanol. Left femurs were frozen in phosphate buffered saline-soaked gauze. All animal procedures were approved by the Indiana University School of Medicine Animal Use and Care Committee (#19004) prior to the initiation of experimental protocols and methods were carried out in accordance with relevant guidelines and regulations.

### Serum biochemistries

Blood collected at time of euthanasia was used to measure serum blood urea nitrogen (BUN) via colorimetric assay to assess the presence of kidney disease (BioAssay Systems, Hayward, CA, USA). Serum 1–84 PTH was measured via ELISA (Immnotopics Quidel, San Diego, CA, USA) which has an intra-assay coefficient of variation of 2.4–5.6%. Serum testosterone was measured via competitive binding ELISA with an intra-assay CV of 2.9–4.0% (R&D Systems, Minneapolis, MN). Serum estradiol was assessed via competitive binding ELISA with an intra-assay CV of 5.4–8.9% (R&D Systems). Serum samples for estradiol were pretreated to remove interfering proteins and protein-bound estradiol as outlined by the manufacturer.

### Ex vivo micro-computed tomography of the femur

Right femurs were scanned on a SkyScan 1172 system (Bruker, Billerica, MA, USA) with a 0.5 aluminum filter and a 6 μm voxel size. Trabecular bone was analyzed in a 1 mm region starting proximal to the growth plate in the distal femur. Cortical bone properties were analyzed on 5 contiguous slices located ~2.5 mm proximal to growth plate in the distal femur. Cortical porosity was determined by assessing void area between the periosteal and endosteal surfaces, presented as a % of overall cortical volume as previously described [15, 16].

### Ex vivo micro-computed tomography of the kidney

Fixed kidneys were scanned on a SkyScan 1176 system (Bruker, Billerica, MA, USA) with no filter and a 9 μm voxel size. A 0.5 mm region of interest was selected in the middle of the kidney (transaxial plane) for assessment of crystal volume/total kidney volume.

### Histomorphometry

Undemineralized right distal femurs were fixed in neutral buffered formalin then subjected to serial dehydration and embedded in methyl methacrylate (Sigma Aldrich, St. Louis, MO). Serial frontal sections were cut 4 μm-thick and left unstained for analysis of fluorochrome calcein labels. Histomorphometric analyses were performed using BIOQUANT (BIOQUANT Image Analysis, Nashville, TN). A standard region of interest of trabecular bone excluding primary spongiosa and endocortical surfaces was utilized. Total bone surface (BS), single-labeled surface (sLS), double-labeled surface (dLS), and interlabel distances were measured at 20x magnification. Mineralized surface to bone surface (MS/BS; [dLS+(sLS/2)]/BS*100), mineral apposition rate (MAR; average interlabel distance/5 days), and bone formation rate (BFR/BS; [MS/BS*MAR]*3.65) were calculated. A second 4 μm-thick section was stained with tartrate resistant alkaline phosphatase (TRAP) for assessment of osteoclasts. TRAP sections were analyzed as osteoclast-covered trabecular surfaces normalized to total trabecular bone surface (Oc.S/BS, %) and then osteoclast surface per unit bone surface within cortical pores (Oc.S/Pore Surface, %). All nomenclature for histomorphometry follows standard usage [17].

### Three-point bending of the femur

Frozen left whole femurs were scanned on a SkyScan 1176 system (Bruker, Billerica, MA, USA) with a 0.5 aluminum filter and a 9 μm voxel size for assessment of mid-shaft cortical parameters. After thawing, samples underwent three-point bending tests with the bottom fixtures positioned 6 mm apart. Femurs were tested until failure with the anterior surface in compression, kept hydrated with phosphate buffered saline, and loaded at a displacement rate of 2mm/min. An 11-lb load cell was utilized for all samples. Using a custom MATLAB script, mechanical outcomes were calculated using force-displacement data and cortical geometry parameters with all mechanical parameters presented using standard nomenclature [18].

### Statistical analysis

Data were analyzed with an unpaired t-test between control and adenine within each sex with no statistical tests comparing sexes together. This approach was chosen rather than a 2x2 factorial (disease-by-sex) since our primary aim was to compare the adenine-induced disease effect between control and adenine mice within each sex, not to determine main and interaction effects of sex with disease. Statistical difference was determined at p<0.05. Partial eta-squared as the effect size (ES) was reported for all values with p<0.05. Linear regression was completed with kidney crystals as the independent factor and serum BUN, serum PTH, and cortical porosity separately as dependent factors. Additionally, another regression analyses was completed with cortical porosity as the dependent factor and PTH as the independent factor. All statistical analyses were completed with IBM SPSS Statistics 26 (IBM, Armonk, NY, USA). Data are presented as mean ± standard deviation.

## Results

### Food intake and body mass

Food intake and body weight during the adenine administration period was lower in both adenine groups with males having approximately 34% lower body weight than control males and females having approximately 22% lower body weight than sex-matched controls after 6 weeks on the adenine diet (S1 Table). Both male and female adenine mice had approximately equivalent food intake throughout the 6-week adenine induction period, ~1.5 gram/day. After the adenine administration period, male adenine mice had a 20% increase in body weight and female adenine mice had a 23% increase in body weight. At study endpoint, male adenine mice weighed 24% lower than controls (p<0.0001, ES = 0.727). Female adenine mice had an average of 8% lower body weight than controls at endpoint (p = 0.02, ES = 0.231; S2 Table).

### Biochemistries and kidney crystals

**Fig 1:**
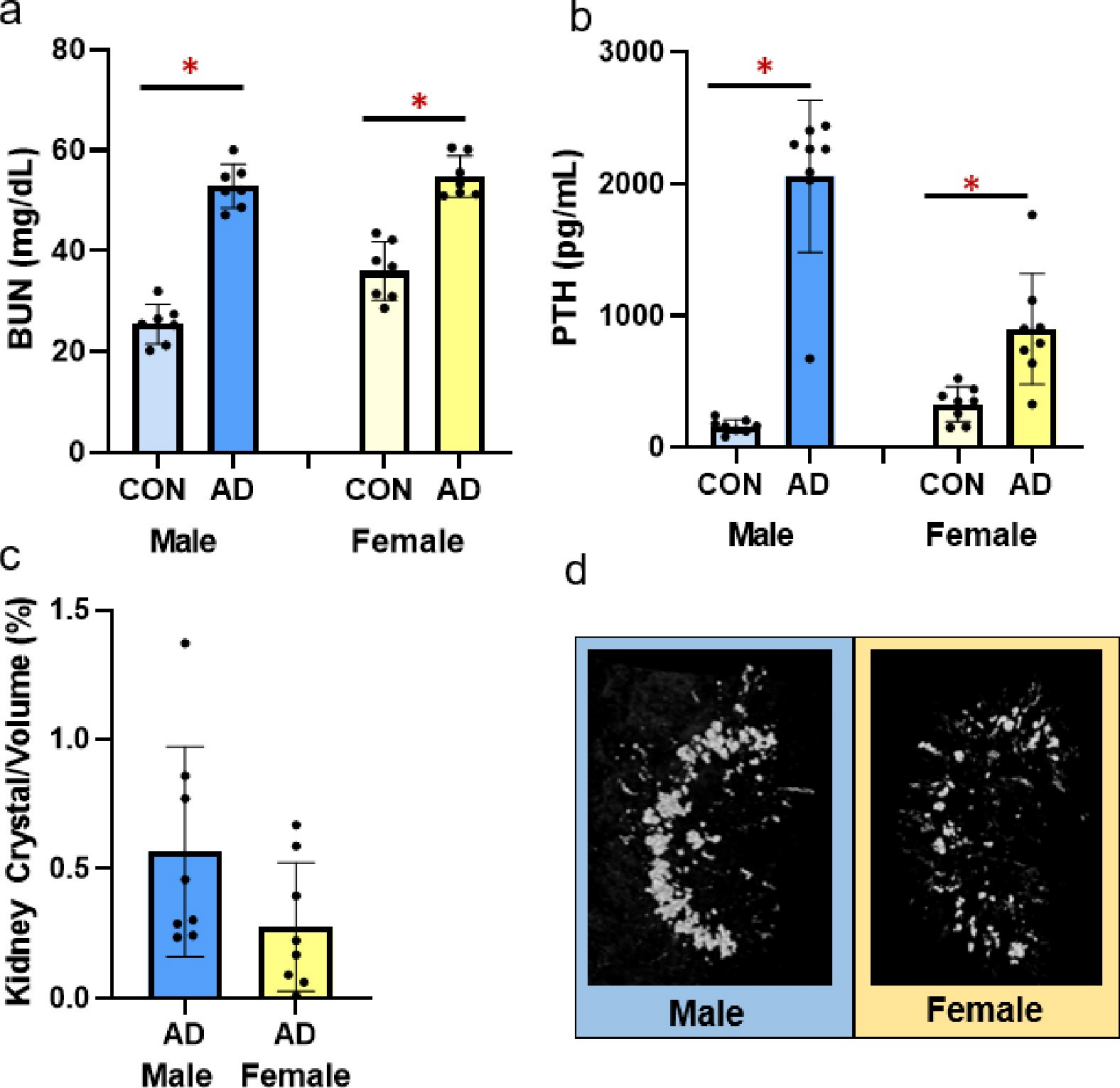
Serum BUN, serum PTH, and adenine kidney crystals in male and female adenine mice. A) Serum BUN was higher in both male and female adenine mice compared to sex-matched controls. B) Serum PTH was higher in adenine-induced CKD in both male and female mice compared to sex-matched controls. C) % kidney crystals present in adenine male and female mice from CT analysis. Control mice had no visible crystals. D) Representative images of kidney crystals in male and female adenine mice. Images represent values closest to the mean of each group. Error bars = standard deviation. *Indicates statistical difference (p<0.05) from t-test between control and adenine within each sex.

At study completion, both male adenine and female mice had higher serum BUN, indicative of CKD, compared to sex-matched controls (*p*<0.0001 for both) with males having a slightly greater disease effect size (ES = 0.927) than females (ES = 0.803; Fig 1A). Adenine-treatment resulted in higher serum PTH in both male (*p*<0.0001) and females (*p* = 0.003) with males have a greater effect size due to disease than females (male ES = 0.860, female ES = 0.491; Fig 1B). Both adenine groups had measurable kidney crystals, measured by CT-based crystal volume/total kidney volume–male adenine mice had 0.56% and female adenine mice had 0.27% (Fig 1C and 1D).

### Serum sex steroids

**Fig 2:**
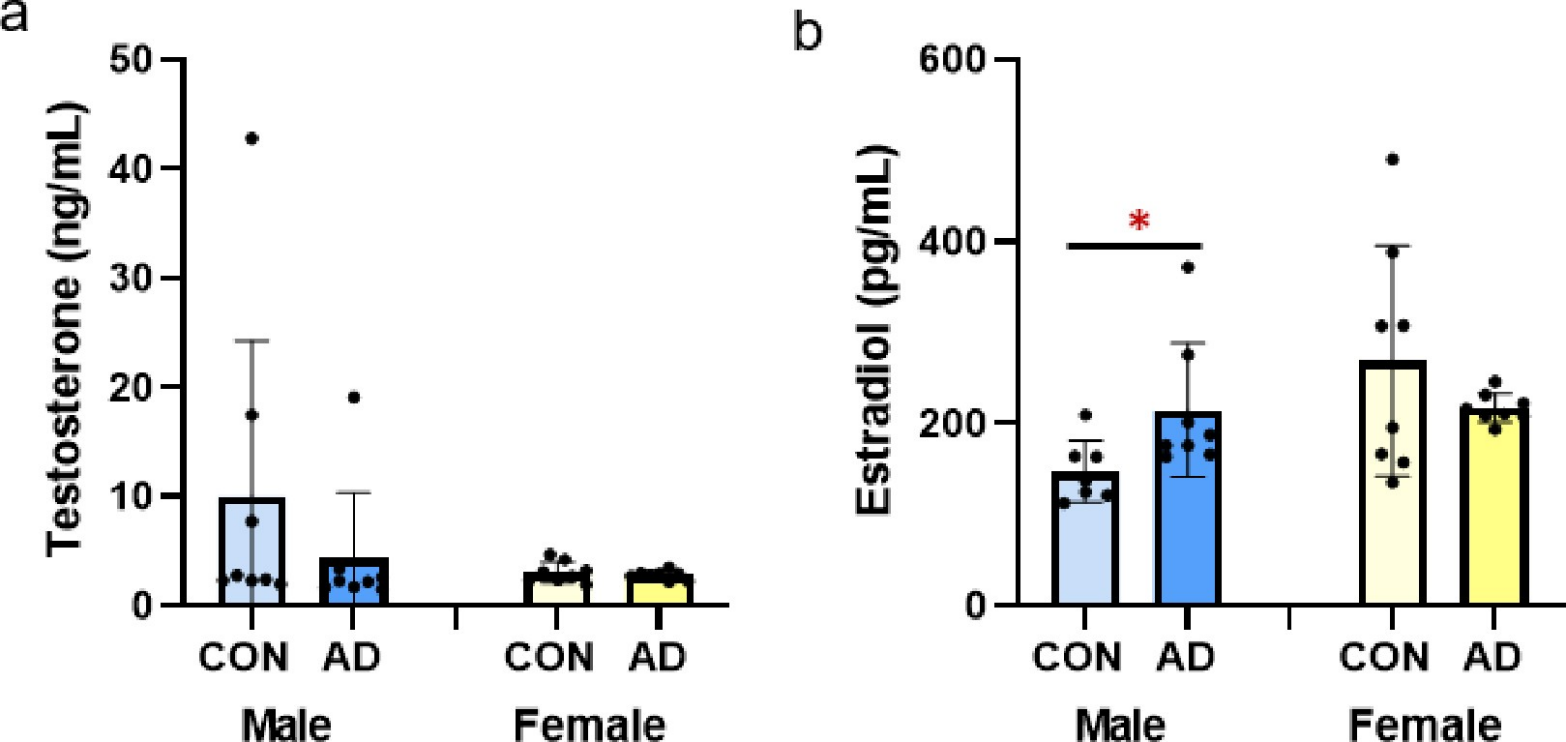
Sex steroids in male and female control and adenine mice. A) Serum testosterone was not different due to adenine in either male or female mice. B) Serum estradiol was higher in male adenine mice vs. control male mice. Estradiol was not different between control and adenine female mice. *Indicates statistical difference (p<0.05) from t-test between control and adenine within each sex.

Serum testosterone was not different due to adenine in either male or female mice (p = 0.318, p = 0.443, respectively; Fig 2A). Serum estradiol was higher in male adenine mice vs. male control mice (p = 0.044, ES = 0.276), but not different between female control and adenine mice (p = 0.274; Fig 2B).

### Cortical bone structure

**Fig 3:**
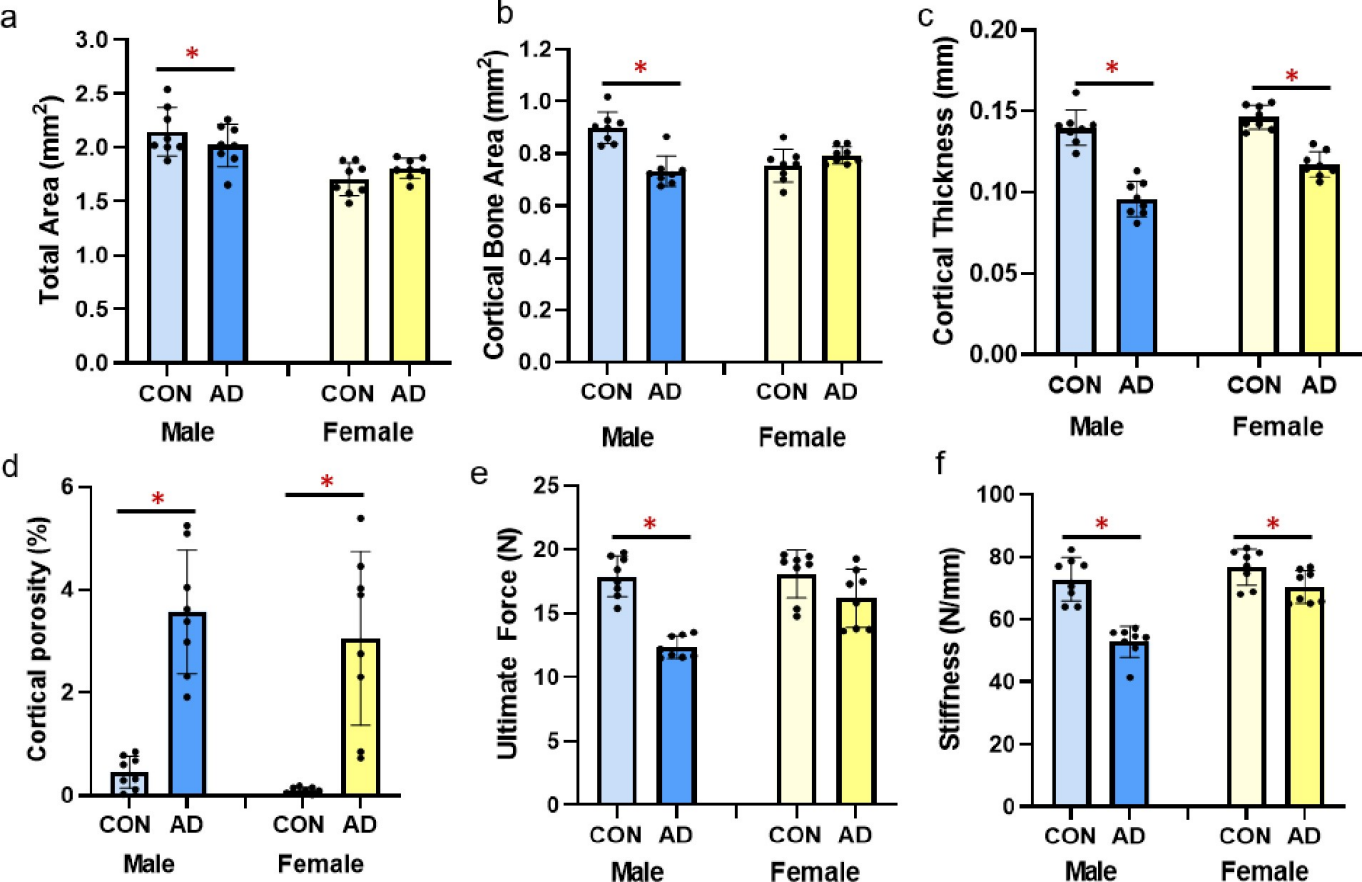
Cortical bone parameters and mechanical properties from the femur of male and female control and adenine mice. A) Total area of the distal 1/3 femur was lower in male adenine mice, but not different in female mice. B) Cortical bone area was lower in male adenine mice with no differences in control vs. adenine in female mice. C) Cortical thickness was significantly lower in both male and female adenine mice. D) Cortical porosity was elevated in both sexes of adenine mice. E) Ultimate force was lower in male adenine mice vs. controls, but not statistically different in female mice. F) Stiffness was lower in adenine mice of both sexes compared to sex-matched controls. Error bars = standard deviation. *Indicates statistical difference (p<0.05) from t-test between control and adenine within each sex.

**Fig 4:**
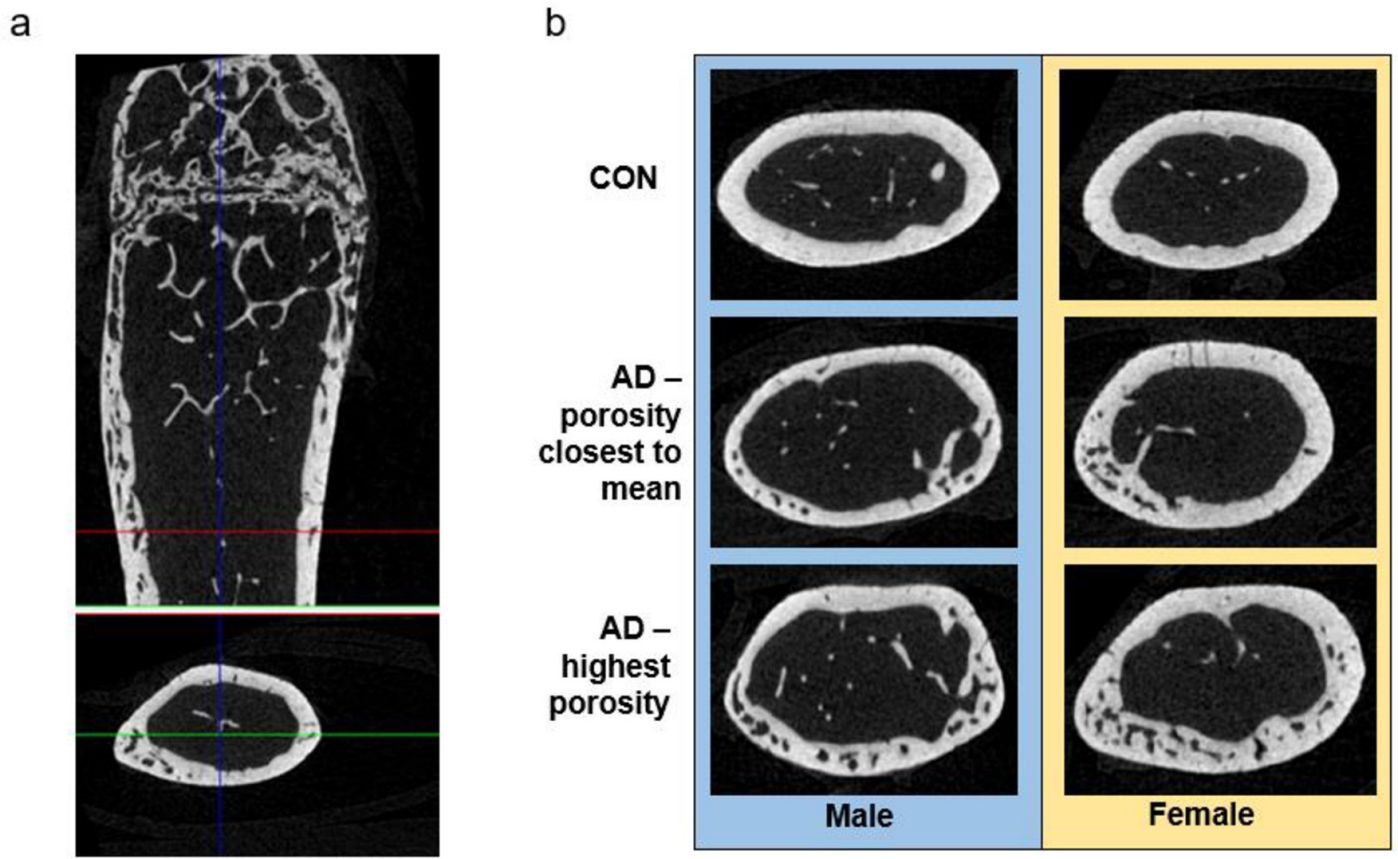
Representative images of cortical bone. A) Representative image from a female adenine mouse of the distal 1/3 region of the femur used for cortical porosity analysis. B) Representative images of cortical sections of controls (closest to mean of porosity), adenine (closest to mean of porosity), and adenine mice with the highest porosity.

Total cross-sectional area of the bone (including bone and marrow space), assessed at ~1/3 total bone length, was not different between control and adenine for either male or female mice (p = 0.251, p = 0.136, respectively; Fig 3A). Cortical bone area was significantly lower in male adenine mice (p<0.0001, ES = 0.691) while not different between female control and female adenine mice (p = 0.131; Fig 3B). In both sexes, cortical thickness was lower in adenine mice compared to matched controls (males p<0.0001, ES = 0.824; females p<0.0001, ES = 0.809; Fig 3C). Significantly higher cortical porosity was present in both male and female adenine mice compared to sex-matched controls (males p<0.0001, ES = 0.783; female p<0.0001, ES = 0.635; Figs 3D and 4).

### Femur mechanics

Male adenine mice had lower ultimate force (p<0.0001, ES = 0.844; Fig 3E) and stiffness (p<0.0001, ES = 0.755; Fig 3F) compared to control male mice. In comparison, female adenine mice only trended towards lower ultimate force (p = 0.09) compared to sex-matched controls but did have lower stiffness vs. controls (p = 0.035, ES = 0.279; Fig 3F). There were not differences in postyield displacement (males p = 0.643, females p = 0.653), total work (males p = 0.205, females p = 0.209), and toughness (males p = 0.571, females p = 0.672) between control and adenine groups for either sex (Table 1).

**Table 1:** Mechanical properties from 3-pt-bend test of the femur.

	Group	Postyield Displacement (mm)	Total Work (mJ)	Toughness (Mpa)
**Male**	*Control*	680.60 ± 606.44	9.82 ± 4.39	2.98 ± 1.36
	*Adenine*	849.69 ± 573.63	7.38 ± 2.79	2.36 ± 0.98
**Female**	*Control*	266.33 ± 157.75	5.92 ± 1.85	2.21 ± 0.89
	*Adenine*	234.23 ± 118.89	4.91 ± 1.11	2.06 ± 0.54

Data represented as mean ± standard deviation. *Indicates statistical difference (p<0.05) from a t-test between control vs. adenine in each sex.

### Trabecular bone structure and histomorphometry

**Fig 5:**
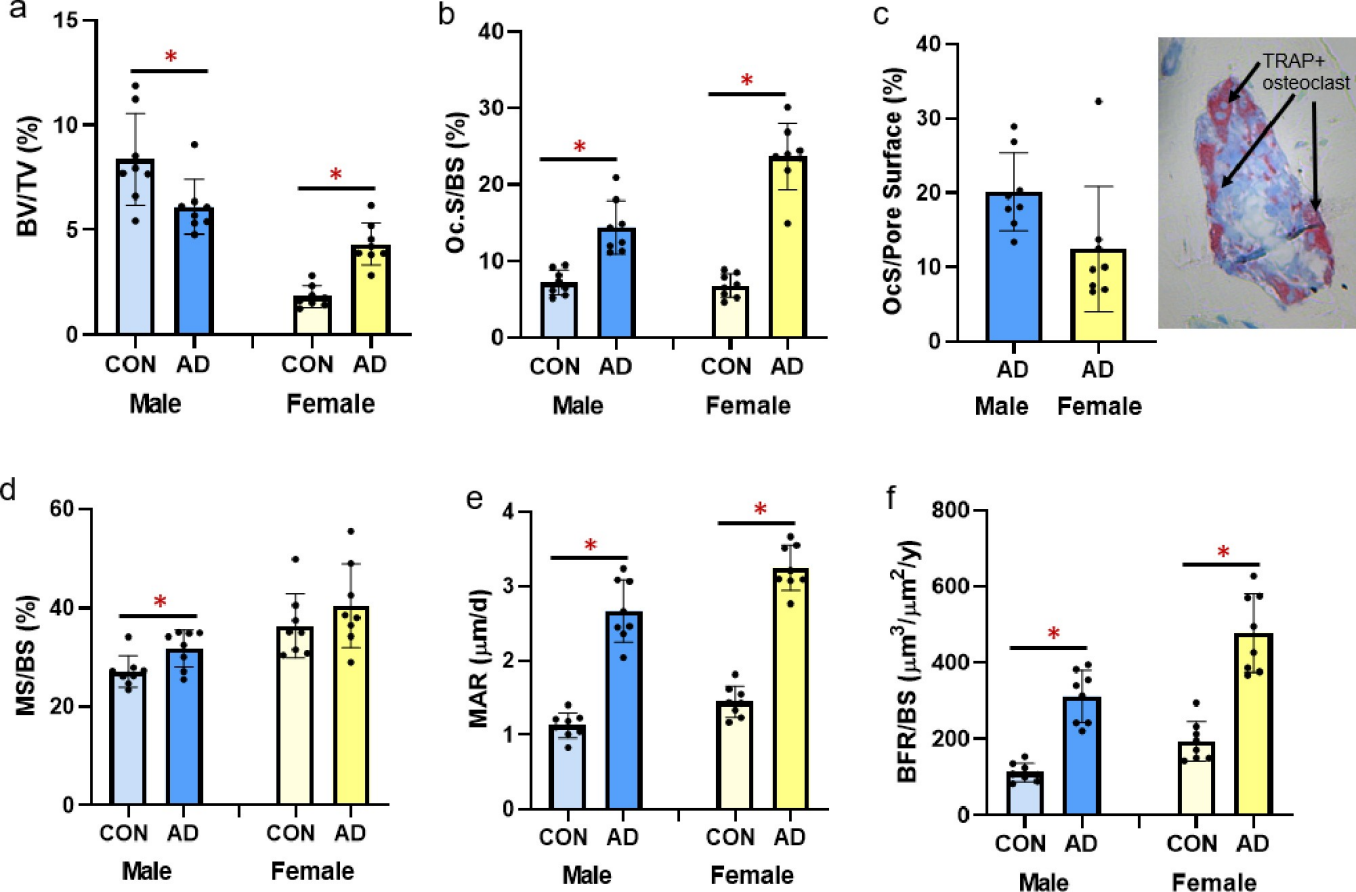
Trabecular bone volume and histomorphometry from the distal femur in male and female B6 mice. A) Trabecular bone volume (%BV/TV) from microCT analysis was statistically lower in male adenine mice compared to male controls, but statistically higher in female adenine mice compared to sex-matched controls. B) Osteoclast-covered trabecular surfaces (Oc.S/BS) were higher in both sexes of adenine mice compared to sex-matched controls. C) Osteoclast-covered pore surfaces in male and female adenine mice and a representative image of a cortical pore in the midshaft femur. D) Mineralized surface (MS/BS) was higher in male adenine mice vs. controls, but not different in female mice. E) Mineral apposition rate (MAR) was higher in both male and female adenine mice compared to sex-matched controls. F) Bone formation rate (BFR/BS) was higher in both sexes of adenine mice. Error bars = standard deviation. *Indicates statistical difference (p<0.05) from t-test between control and adenine within each sex.

Male adenine mice had lower trabecular BV/TV (p = 0.024, ES = 0.313; Fig 5A), lower trabecular thickness (p = 0.036, ES = 0.278), higher trabecular separation (p<0.0001, ES = 0.754), and lower trabecular number (p = 0.04, ES = 0.267; Table 2) compared to sex-matched controls. In contrast, female adenine mice had higher BV/TV (p<0.0001, ES = 0.735; Fig 5A) and higher trabecular number (p<0.0001, ES = 0.845; Table 2) compared to sex-matched controls. In female mice, there were not differences between control and adenine in trabecular thickness (p = 0.113) or trabecular separation (p = 0.157).

**Table 2:** Trabecular parameters from microCT of the distal femur.

	Group	Tb.Th (mm)	Tb.Sp (mm)	Tb.N (#/mm)
**Male**	*Control*	0.05 ± 0.00	0.24 ± 0.01	1.70 ± 0.34
	*Adenine*	0.04 ± 0.00	0.30 ± 0.02*	1.40 ± 0.16*
**Female**	*Control*	0.03 ± 0.01	0.33 ± 0.01	0.52 ± 0.08
	*Adenine*	0.04 ± 0.00	0.34 ± 0.02	1.08 ± 0.16*

Data represented as mean ± standard deviation.

*Indicates statistical difference (p<0.05) from a t-test between control vs. adenine in each sex.

Osteoclast surface/bone surface was statistically higher in male (p<0.0001, ES = 0.664) and female (p<0.0001, ES = 0.884; Fig 5B) adenine mice compared to sex-matched controls. Assessment of cortical pore surfaces covered by osteoclasts found that 20% (SD = 5.2) and 12% (SD = 8.4) of pore surfaces were covered by osteoclasts in both sexes of adenine mice, respectively (Fig 5C). Male adenine mice had higher mineralized surface to bone surface (MS/BS; p = 0.019, ES = 0.336; Fig 5D), higher mineral apposition rate (MAR; p<0.0001, ES = 0.868; Fig 5E) and higher bone formation rate (BFR/BS; p<0.0001, ES = 0.812; Fig 5F) compared to sex-matched controls. Female adenine mice also had higher MAR (p<0.0001, ES = 0.931; Fig 5E) and BFR/BS (p<0.0001, ES = 0.777; Fig 5F) while MS/BS was not different (p = 0.297; Fig 5D).

### Linear regression

**Fig 6:**
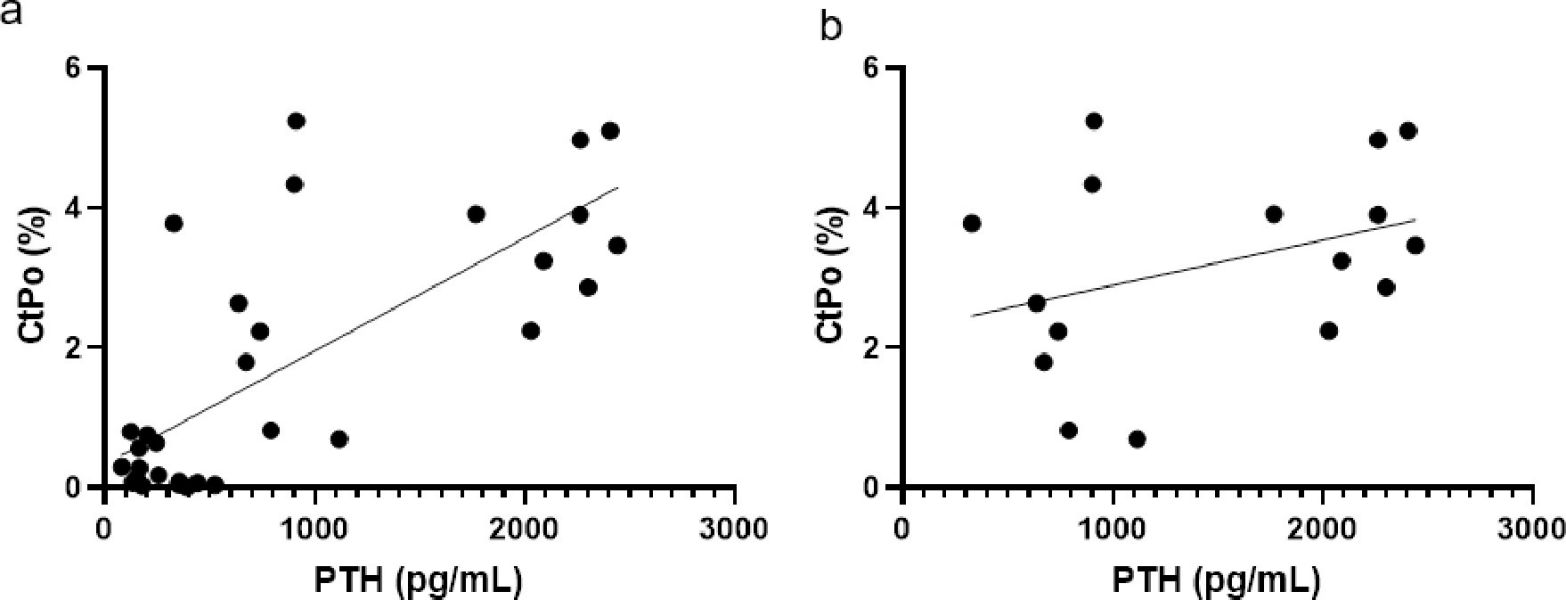
Linear regression plots of PTH to cortical porosity. A) Regression plot of all male and female mice, control and adenine. R^2^ = 0.559. B) Regression plot of only male and female adenine mice. R^2^ = 0.125.

With only adenine mice of both sexes, kidney crystals did not predict serum BUN (R^2^ = 0.251, p = 0.068) or cortical porosity (R^2^ = 0.099, p = 0.274)), but did significantly predict serum PTH levels (R^2^ = 0.403, p = 0.015). With both sexes of control and adenine mice combined, linear regression analyses demonstrated that PTH statistically predicated the variability in cortical porosity (R^2^ = 0.559, p<0.0001; Fig 6A). With only the adenine mice included, there was no statistical relationship between PTH and cortical porosity (R^2^ = 0.125, p = 0.178; Fig 6B).

## Discussion

The primary finding of this study is that both male and female C57Bl/6J mice develop skeletal complications due to adenine-induced CKD, including high bone turnover, cortical thinning, and development of cortical porosity. These phenotypes are associated with elevated PTH in both sexes. Male mice with adenine-induced CKD appear to have a modestly more severe response with significant reductions in cortical bone area and notable changes to key mechanical properties such as ultimate load.

Declines in kidney function, the driving force of CKD, are associated with reduced renal clearance of circulating substances including blood urea nitrogen. In adenine-induced CKD, BUN is elevated within two weeks of introducing adenine into the diet of mice and remains high [15]. In our current study, serum BUN was elevated in all adenine mice compared to sex-matched controls indicating the presence of kidney disease. Previous studies have shown that adenine ingestion causes tubulointerstitial damage resulting in consistent and stable renal impairment in mice [19]. Previously, we quantified adenine-induced kidney crystals over time in young female mice and found kidney crystals were only notable 10 weeks after diet initiation [15]. In this study, kidney crystals were present in both sexes of adenine mice with males having a higher prevalence of kidney crystals than females. Interestingly, both sexes of adenine mice had similar food intake while on the adenine diet indicating the amount of crystals present via CT analysis may not be directly associated with adenine ingestion. It appears plausible that kidney crystals and/or location of crystals may be associated with disease severity possibly due to greater 2,8-dihydroxyadenine precipitation indicating a more severe disease in males vs. females; however, linear regression showed no relationship between kidney crystals and BUN or cortical porosity although ~40% of the variability in PTH was predicted by kidney crystals. The influence of kidney crystals on disease progression in this model is not fully understood; however, we and others have consistently shown reductions in renal function in mice with adenine-induced CKD and features consistent with human chronic kidney disease-mineral bone disorder [15, 16, 19, 20].

Disruptions in mineral homeostasis due to CKD often lead to secondary hyperparathyroidism which is associated with cortical porosity in humans [1] and in rodent models [15, 21]. In male mice, adenine-induced CKD has been shown to create kidney disease with high PTH [22]. In a separate study, female mice with adenine-induced CKD also had high PTH as well as lower bone mineral density and cortical thinning [23]. Overall, our data indicate that both male and female mice with the same induction paradigm of adenine-induced CKD develop high PTH and have lower cortical bone through cortical thinning and high cortical porosity. Males appear to be slightly more sensitive to CKD disruptions with a greater effect size for PTH and less variability in cortical porosity compared to females. For cortical porosity, male adenine mice had an average of 3.5% porosity with a range from 1.9% to 5.2% while females had an average porosity of 3% with a range from 0.7% to 5.4%. Linear regression analyses with all control and adenine mice demonstrated that approximately 56% of the variability of cortical porosity was predicted by endpoint PTH values; however, there is no statistical relationship between PTH and cortical porosity with only adenine mice. Therefore, PTH is clearly an important factor for cortical porosity development in CKD but, once elevated, may not be directly related to the amount of porosity, although our study only assessed a single site of cortical porosity.

While the role of sex steroids on bone health, particularly the protective role of estrogen and the impact of the loss of estrogen, are well documented, the role of sex steroids in CKD are less understood. Generally, testosterone is considered to be deleterious in CKD while estrogen is renoprotective [24]. In rats with adenine-induced CKD, adenine reduced serum testosterone in males, but had no impact on 17β-estradiol in females [14]. In another study with adenine-induced CKD rats, male adenine rats had lower testosterone and higher estrogen compared to control rats while female rats had no differences in sex steroids [25]. In our study, male adenine mice had a non-statistically significant 55% lower serum testosterone and 45% higher serum estradiol than control male mice while adenine had no impact on sex steroids in female mice. Overall, these changes were minor compared to changes in PTH which we hypothesize is a more prominent driver of the skeletal changes in this model; however, the greater differences in sex steroids in male mice could contribute to the more severe skeletal phenotype.

Besides the presence of cortical pores, both male and female mice with adenine-induced CKD had lower cortical thickness with males having a greater effect size due to adenine (31% lower) than females (21% lower compared to controls). Male mice had ~6% lower total area in adenine vs. control as well as 22% lower cortical bone area while female mice did not have statistical differences in these measures. Overall, these changes indicate a more severe cortical bone phenotype in male mice with adenine-induced CKD vs. female mice.

Like the sex-specific changes in cortical bone structure, mechanical changes due to adenine-induced CKD were more notable in male vs. female mice. Male adenine mice had a deficit in ultimate force vs. sex-matched controls (31% lower) while female adenine mice had a 10% difference between control and adenine that did not reach statistical difference. Likewise, there was a greater difference in stiffness in male adenine mice than in female adenine mice compared to their sex-matched non-adenine controls (27% lower and 8% lower, respectively). Male adenine mice also weighed significantly less compared to control counterparts than did female mice which could contribute to the more notable alterations in mechanical properties. In clinical studies, both sexes have an increasing risk of fracture with increasing stages of CKD; however, females have a higher rate of fracture than males especially with the combination of age and CKD [26]. There are likely other confounding factors at play in clinical studies, such as post-menopausal or aging-induced bone loss, that may interact with CKD; future studies should consider incorporating these factors into the adenine model to dissect out contributions.

In CKD patients, trabecular bone is often spared while cortical bone is preferentially lost through the development of cortical porosity [27]. Over a one-year period, patients with CKD had rapid increases in cortical porosity and cortical thinning measured via HR-pQCT at the distal radius while trabecular bone remained largely unchanged [1]. In a cross-sectional study, female end-stage renal disease patients had greater deficits in distal tibia trabecular bone volume compared to matched controls than did males indicating a sex-specific differences in trabecular bone response [28]. Results from this current study showed that male adenine mice had 27% lower trabecular bone volume than matched controls, but female adenine mice had 97% higher trabecular bone volume than matched controls. Both sexes of adenine mice had elevated bone formation rate due largely to increased mineral apposition rate (~2.2-fold higher compared to matched controls) indicating increased activity of osteoblasts. Additionally, male adenine mice had 2-fold higher osteoclast-covered trabecular surfaces and female adenine mice had 3.5-fold higher osteoclast-covered surfaces compared to sex-matched controls. Since both males and females had high bone formation and high osteoclast surfaces indicating high turnover, we are uncertain of the cause of higher trabecular bone volume in female adenine mice. The higher trabecular bone volume in female mice could be due to sex-specific differences in the response to adenine over time as our study only measured bone turnover in the last week of the study. For example, female mice could have greater formation with lower resorption in an earlier phase than male mice resulting in higher BV/TV. It is also possible there are some adenine-specific skeletal effects. For example, male Wistar rats on adenine had greater trabecular thickness and bone formation rate than 5/6 nephrectomy counterparts despite no differences in serum creatinine or PTH [29].

It is important to note that clinically CKD can manifest as normal, high, or low bone turnover. For example, bone biopsy analysis of pre-dialysis and dialysis CKD patients demonstrated 31% of pre-dialysis patients had high turnover while 50% of dialysis patients had high turnover [30]. Another study found racial differences with 62% of the White population studied had low turnover and 68% of the Black population had normal-to-high turnover [31]. Additionally, serum PTH levels correlate with high bone turnover and more prevalent cortical porosity [31]. The adenine-induced CKD model represented here mirrors the high PTH/high bone turnover manifestation of CKD as do other animal models [10, 29]. Therefore, adenine-induced CKD in mice models a specific bone phenotype within the complexity of clinical manifestations of CKD.

Limitations of our current studies are the use of only one time point; therefore, we cannot determine the time course of development of changes in the mice and thus the potential exists for phenotypes in the female to simply take longer to develop. We also did not do extensive analyses of sex steroid hormones and their receptors, a potential factor driving the modest differences between sexes–future work could focus on analyses such as the role of estrogen receptors in CKD bone. Finally, while the current study is examining CKD, our laboratory is focused on the skeletal manifestations of the disease and thus did not undertake assessments of kidney function/pathology beyond basic measures of BUN, which we used as a basic marker of altered kidney function.

In conclusion, we show that both male and female C57Bl/6 mice develop a high bone turnover phenotype with adenine-induced CKD characterized by elevated PTH, cortical thinning, and cortical porosity. Unlike some other animal models of CKD, both sexes developed a similar phenotype; however, males have a slightly more severe skeletal response with greater effects on cortical bone and mechanical properties than females. These data support the use of the adenine-induced CKD model in C57Bl/6 mice for studying sex as a biological variable on skeletal changes and interventions in CKD.

## Supporting information

S1 Table Food intake. Average daily food intake (g) per mouse per cage. Gray denotes 6-week period when mice were on adenine diet. Statistical analyses were not completed due to low n (2–3 cages per group).
(JPG)

S2 Table Body weight. Average body weight (g) over time. *p<0.05 between control and adenine within each sex.
(JPG)
