# Supplementary figures and images for: Adenine-induced chronic kidney disease induces a similar skeletal phenotype in male and female C57BL/6 mice with more severe deficits in cortical bone properties of male mice
Source: PLoS One. 2021 Apr 23;16(4):e0250438. doi: 10.1371/journal.pone.0250438 (PMC8064570; doi:10.1371/journal.pone.0250438)

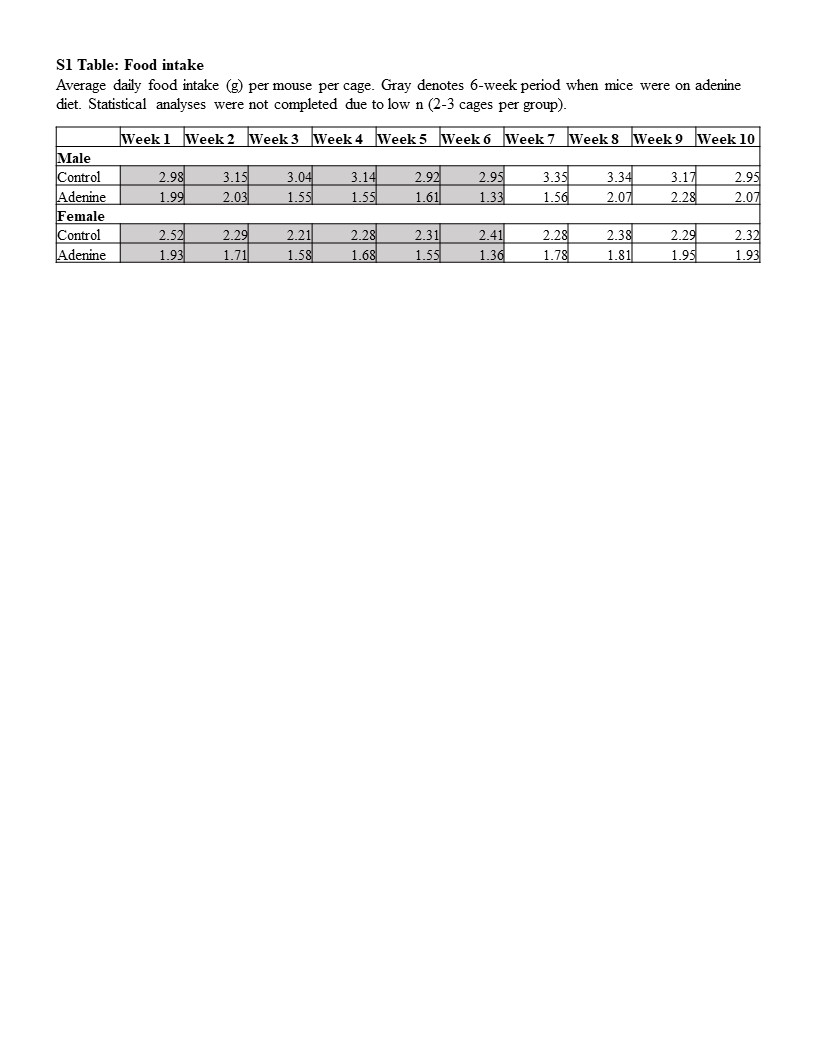

Supplement: S1 Table — Average daily food intake (g) per mouse per cage. Gray denotes 6-week period when mice were on adenine diet. Statistical analyses were not completed due to low n (2–3 cages per group). (JPG) [file pone.0250438.s001.JPG]

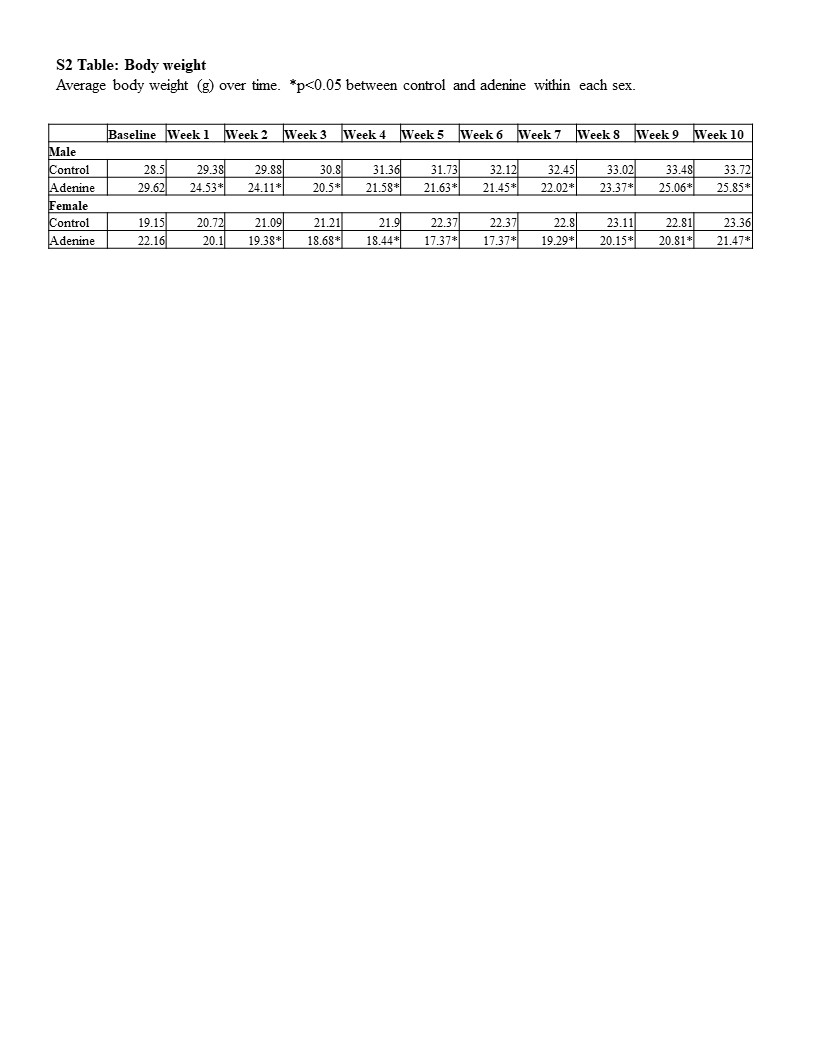

Supplement: S2 Table — Average body weight (g) over time. *p<0.05 between control and adenine within each sex. (JPG) [file pone.0250438.s002.JPG]
